# Clinical Evaluation of a Royal Jelly Supplementation for the Restoration of Dry Eye: A Prospective Randomized Double Blind Placebo Controlled Study and an Experimental Mouse Model

**DOI:** 10.1371/journal.pone.0169069

**Published:** 2017-01-06

**Authors:** Sachiko Inoue, Motoko Kawashima, Ryuji Hisamura, Toshihiro Imada, Yusuke Izuta, Shigeru Nakamura, Masataka Ito, Kazuo Tsubota

**Affiliations:** 1 Department of Ophthalmology, Keio University School of Medicine, Shinjuku-ku, Tokyo, Japan; 2 Haneginomori Eye Clinic, Daita, Setagaya-ku, Tokyo, Japan; 3 Developmental Anatomy and Regenerative Biology, National Defense Medical College, Tokorozawa, Saitama, Japan; Xiamen University, CHINA

## Abstract

**Background:**

Dry eye is a multifactorial disease characterized by ocular discomfort and visual impairment. Lacrimal gland function has been shown to decrease with aging, a known potent risk factor for dry eye. We have previously found that orally administrated royal jelly (RJ) restored tear secretion in a rat model of dry eye.

**Methods and Findings:**

We examined the effects of RJ oral administration on dry eye in this prospective, randomized, double-blind, placebo-controlled study. Forty-three Japanese patients aged 20–60 years with subjective dry eye symptoms were randomized to an RJ group (1200 mg/tablet, six tablets daily) or a placebo group for 8 weeks. Keratoconjunctival epithelial damage, tear film break-up time, tear secretion volume, meibum grade, biochemical data, and subjective dry eye symptoms based on a questionnaire were investigated at baseline, and at 4 and 8 weeks after intervention. Adverse events were reported via medical interviews. In the RJ group, tear volume significantly increased after intervention (*p* = 0.0009). In particular, patients with a baseline Schirmer value of ≤10 mm showed a significant increase compared with baseline volume (*p* = 0.0005) and volume in the placebo group (*p* = 0.0051). No adverse events were reported. We also investigated the effect of RJ (300 mg/kg per day) administration using a mouse model of dry eye. Orally repeated administration of RJ preserved tear secretion, potentially through direct activation of the secretory function of the lacrimal glands.

**Conclusion:**

Our results suggest that RJ improves tear volume in patients with dry eye.

**Trial Registration:**

Registered NO. the University Hospital Medical Information Network in Japan (UMIN000014446)

## Introduction

Dry eye is a common disease and recognized as a growing public health problem that degrades daily quality of life [1, 2] It is a multi-factorial disease characterized by ocular discomfort and visual impairment. The function of the lacrimal glands (LGs), which secrete tears, has been shown to decrease with aging [3–5]. Furthermore, the use of digital devices is also a risk factor for dry eye [6]. The incidence of this condition has been increasing in the ageing modern society, including Japan [1].

Beekeeping products have been deeply rooted as nutritional sources and medicines in the lives of different people and cultures worldwide for thousands of years. Honeybee products are used as a nutritional source in daily life and medicine [7–13]. The main products of beekeeping include honey, royal jelly (RJ), propolis, pollen, and bee larva produced or collected by Apidae. The efficacy of these products in the field of ophthalmology has been demonstrated [14,15].

RJ, which the queen honeybee requires for its development, is mainly secreted by the mandibular and hypopharyngeal glands of worker honeybees (*Apis mellifera*). RJ is a complex substance comprising proteins, sugars, lipids, amino acids, vitamins, and minerals [9]. Previously, our research group found that orally administered RJ restored tear secretion capacity in a dose-dependent manner in a rat model of dry eye [16]. We conducted this randomized, double-blind, placebo-controlled trial to investigate the effects of RJ on dry eye signs and symptoms in human patients. In addition, we investigated underlying lacrimal mechanisms in relation to the results of this human study using a mouse model of dry eye.

## Materials and Methods

### Human study

The protocol for this trial and supporting CONSORT checklist are available as supporting information; see S1 Text CONSORT Checklist, S2 Text Protocol in Japanese, and S3 Text Protocol in English.

#### Ethics

This study adhered to the guidelines of the Declaration of Helsinki (amended in 2008). The study protocol was approved by the Shirasawa Clinical Research Center Ethical Review Board (approved No. 214004). All subjects received a full explanation of the study procedures, and written informed consent was obtained from all prior to enrollment. To ensure privacy, all records were identified by an anonymous subject identification number.

#### Study design

This study was a prospective, randomized, placebo-controlled, parallel-design trial designed to assess the efficacy and safety of orally supplemented RJ in patients with subjective symptoms of dry eye through ophthalmological examinations (objective) and a questionnaire (subjective). The study was conducted between July 2014 and September 2014 at the Haneginomori Eye Clinic, Tokyo, Japan and is registered with the University Hospital Medical Information Network in Japan (UMIN000014446).

#### Subjects

Japanese men and women aged 20–60 years who complained of subjective symptoms of dry eye, such as dry eye sensation and foreign body sensation, were recruited. The exclusion criteria were as follows: current or previous severe ocular diseases such as strabismus, cataract or glaucoma; a risk of developing seasonal allergy between July and September; laser-assisted in situ keratomileusis surgery within the previous 3 months; and allergy to the test supplement. Patients were also excluded if they routinely used multi-vitamin or mineral supplements, or if they were currently taking medicines and/or receiving chronic medical treatment (including prescribed eyedrops) to improve their vision and ocular symptoms. All patients were permitted to withdraw from the study at any time for any reason. Additional exclusion criteria were as follows: a history of any other serious disease requiring medical treatment; participation in another clinical trial within 1 month prior to the start of the present study; and pregnancy or lactation during the study period.

#### Ophthalmological examinations

The tear film break-up time (BUT) was measured, keratoconjunctival epithelial damage based on fluorescein staining scores for the cornea, the and conjunctiva was assessed by two experienced investigators (M.K. and S.I.) using previously described methods [17]. For these examinations, after 2 μl of preservative-free, 1% fluorescein dye was instilled into each eye, BUT was measured three times, and the mean value was analyzed. The cornea and the conjunctiva fluorescein staining scores were graded from 0 (none) to 3 (severe) [17,18]. Meibomian gland secretions (meibum grade) were semi-quantitatively evaluated as follows: grade 0, clear, easily expressed meibum; grade 1, cloudy meibum expressed with mild pressure; grade 2, cloudy meibum expressed with more than moderate pressure; and grade 3, no meibum expression, even with hard pressure [19]. The tear volume was evaluated using the Schirmer test I, which was performed for 5 min without anesthesia. These ocular examinations were performed at baseline, and 4 and 8 weeks after intervention.

#### Diagnosis of dry eye

A dry eye diagnosis was made according to the latest Japanese dry eye diagnostic criteria (2006) [18]. Briefly, 1) presence of dry eye symptom; 2) presence of qualitative or quantitative disturbance of the tear film (Schirmer test ≤5 mm or BUT ≤5 seconds); and 3) presence of keratoconjunctival epithelial damage (total score of fluorescein staining ≥3 points). A diagnosis of definite dry eye required adherence to all the three criteria. Subjects fulfilling two of the three criteria were diagnosed as having probable dry eye, and those fulfilling one or none of the three criteria were defined as having non-dry eye.

#### Functional visual acuity measurements

An functional visual acuity (FVA) measurement system (Kowa, Aichi, Japan) was used to assess the time course of changes in the continuous visual acuity (VA) for 1 min. In this method, the Landolt optotypes are presented on the monitor, and their sizes change depending on the correctness of the responses [20,21]. The average FVA was measured at baseline, and 4 and 8 weeks after intervention.

#### Assessment of subjective symptoms self-reported via a questionnaire

The validated dry eye symptom questionnaire: the Dry Eye-Related Quality-of-Life Score (DEQS) questionnaire was administered at baseline, and 4 and 8 weeks after intervention [22]. The DEQS questionnaire comprises 15 questions; six questions assess the ocular symptoms and nine assess the effect of dry eye disease on the quality of life. The six questions related to ocular symptoms query respondents on the presence and severity of foreign body sensations, dry sensations, pain or soreness, ocular fatigue, eyelid heaviness, and eye redness. The frequency of symptoms is scored from 0 (none) to 4 (highest frequency) and the severity of symptoms is scored from 1 (low) to 4 (high).

#### Serum biochemical analysis

Biochemical assessments (total protein, aspartate aminotransferase, alanine transaminase, lactate dehydrogenase, alkaline phosphatase, gamma-glutamyl transferase, urea nitrogen, creatine, uric acid, total cholesterol, low-density lipoprotein cholesterol, high-density lipoprotein cholesterol triglyceride, glucose, and hemoglobin A1c) were performed at baseline and 8 weeks after intervention.

#### Interventions

Patients were randomly allocated to be administered placebo tablets or RJ tablets (400 mg enzyme-treated royal jelly/tablet; six tablets/day; Yamada Bee Company, Inc., Tomata-gun, Okayama, Japan) for 8 weeks. The enzyme-treated royal jelly was standardized to contain minimum 3.5% (E)-10-hydroxy-2-decenoic acid and minimum 0.6% 10-hydroxydecanoic acid. The placebo tablets were colored similarly to the RJ tablets, and both tablet types were packaged in an opaque bag to ensure blinding. All eligible patients were randomly assigned (1:1) to receive RJ (RJ group) or placebo (control group) tablets. Two tablets were to be ingested with sufficient water three times daily (total six tablets) after each meal for 8 weeks. Patients were instructed to self-record their allocated capsule intake status and any adverse events in a study diary for the entire intervention period. During the trial, patients were instructed to maintain lifestyle habits from before the trial in areas such as diet, alcohol consumption, exercise, and sleep. In addition, they were instructed to avoid large deviations from the normal range for exercise and food consumption (both under and over-eating), and not to allowed to consume any new health foods or supplements during the trial. The use of artificial tears was permitted up to four times daily for participants who used artificial tears before entry in the study.

### Safety assessments

Patients were instructed to report any adverse events during a medical interview conducted at every visit during the study period. Biochemical data were also used for safety assessments.

#### Statistical analysis

The primary outcomes were dry eye parameters. We planned a study of continuous response variables in matched pairs of study subjects. Prior data indicated that the difference in the responses of matched pairs was normally distributed with standard deviations. If the true difference in the mean responses of matched pairs was five, we would be required to study 17 pairs of subjects to be able to reject the null hypothesis that the difference in responses is zero with a probability (power) of 0.8. The Type I error probability associated with testing of this null hypothesis was 0.05.

For the baseline characteristics of patients with dry eye, Student’s *t*-test (*p* ≥ 0.05 by the F-test) was used for comparison of age, and Pearson chi-square test was used for categorical data of gender and dry eye diagnosis. Repeated measures analysis of variance followed by Tukey's honest significant difference (HSD) post hoc test was used for analysis of dry eye symptoms. For all parameters, the between-group differences (RJ vs placebo) were assessed using the Aspin–Welch test or Student’s *t*-test after the F-test. For the biochemical analysis, paired *t*-tests were used for within-group comparisons and the F-test, followed by Student’s *t*-test (*p*≥0.05 by the F-test), were used for between-group comparisons (placebo vs. RJ). A *p*-value of <0.05 was considered statistically significant. All statistical analyses were performed using the PASW Statistics 18 (SPSS Japan, Tokyo, Japan) program and JMP 8.0 (SAS Institute, Cary, North Carolina, USA).

### Animal study

Tear secretion is regulated by the coordination between the LGs and autonomic nervous system activity [23]. To clarify whether orally administrated RJ directly activates the secretory capacity of LGs, as opposed to the autonomic nervous system, we assessed the effects of RJ using a mouse LG postganglionic denervation (PGD) dry eye model. This created a condition whereby neuronal stimuli from the central nervous autonomic system were interrupted.

#### Animals

Female C57BL/6 mice (CLEA Japan, Japan) aged 8 weeks were used in the present study. All animals were quarantined and acclimatized for a week prior to the experiments under the following general conditions: room temperature, 23 ± 2°C; relative humidity, 60% ± 10%; alternating 12-h light–dark cycle (8 AM to 8 PM); and free access to water and food. All mice were treated in accordance with the Ethics Committee on Animal Research of the Keio University School of Medicine (Approval No. 12111–1) and the Association of Research and Vision in Ophthalmology (ARVO) statement for the Use of Animals in Ophthalmic and Vision Research.

#### Postganglionic denervation dry eye model

The mice underwent PGD or sham denervation (Sham) surgery. After mice were deeply anesthetized with sodium pentobarbital (64.8 mg/kg, intraperitoneal [i.p.]), the nerve bundle was detached from the blood vessels at the caudal root site of the ventral surface of the LGs. A nylon thread was hung on this nerve bundle, and both ends of the thread were passed inside the polyethylene tube. The nerve bundle was ligated by pulling these ends.

#### Royal Jelly application

RJ was collected from North and East China. Protease-digested lyophilized powder of RJ (enzyme-treated RJ) was supplied by Yamada Bee Company, Inc. (Tomata-gun, Okayama, Japan). The enzyme-treated royal jelly was standardized to contain minimum 3.5% (E)-10-hydroxy-2-decenoic acid and minimum 0.6% 10-hydroxydecanoic acid. RJ was dissolved in distilled water and repeatedly administered orally once daily after LG denervation. The dose was set at 300 mg/kg per day, according to a previously reported effective dosage in a murine study [16]. In the Sham group, distilled water was repeatedly administered orally once a day. Six mice were used in each group.

#### Measurements of tear secretion

We used a modified Schirmer test on mouse eyes to measure basal tear secretion [6]. A phenol red thread (Showa Yakuhin Kako, Japan) was placed on the temporal side of the upper eyelid margin for 15 s. To alleviate suffering, the measuring time was shortened to 15 s in this mouse model compared with 5 min in humans. The length of the moistened area from the edge was measured within 0.5 mm.

#### Histopathological examination

Mice were sacrificed by exsanguination through cutting the carotid artery, after being deeply anesthetized with sodium pentobarbital (120 mg/kg, i.p.). The LGs of each animal were removed after death and immediately fixed in 10% formalin. The LGs were then embedded in paraffin and sectioned. Sections were subjected to hematoxylin and eosin staining.

#### Transmission electron microscopy

Each mouse was perfused with Karnovsky’s fixative (2.5% glutaraldehyde and 2% paraformaldehyde in 0.1 M sodium cacodylate; pH 7.4) under deeply anesthetized with sodium pentobarbital (64.8 mg/kg, i.p.). After the LGs were removed from the bodies, they were immersed in the same fixative at 4°C. The ultrathin sections were examined under a transmission electron microscopy (1200 EXII, JEOL, Japan).

## Results

### Human study

#### Baseline patient characteristics

**Fig 1:**
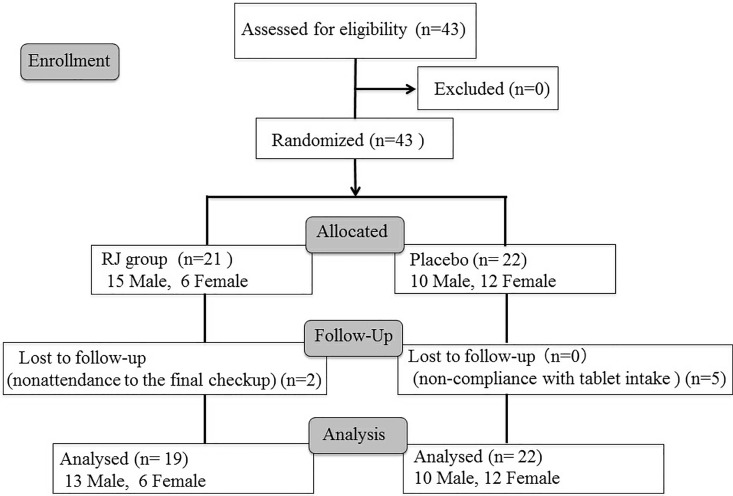
CONSORT flowchart.

Forty-three patients were enrolled in this intervention study and assigned to either the placebo (n = 22) or RJ group (n = 21). None of the patients had severe dry eye, such as in Sjogren's syndrome. Two patients were excluded from the RJ group due to being lost to follow-up. Finally, 41 patients were analyzed, 22 and 19 in the placebo and RJ groups, respectively (Fig 1). Table 1 shows the baseline characteristics of patients in both groups. The mean age in all patients was 33.3 ± 9.18 years (range, 21–53 years). The mean age in the RJ group was 29.4 ± 8.26 (range, 21–49 years) and 37.0 ± 8.63 years (range, 23–53 years) in the placebo group. The values ware statistically significant between RJ group and placebo group (*p* = 0.0054). There were no significant differences in any of the other variables between the two groups.

**Table 1:** Baseline characteristics of patients with dry eye who received placebo (PB) or royal jelly (RJ).

Characteristic	Total	RJ group	PB group	*p*-value
Number of subjects	43	21	22	
Age (mean ± SD, range)	33.30 ± 9.18 (21–53)	29.57 ± 8.26 (21–49)	37.00 ± 8.63 (23–53)	0.0054
Men (number; %)	25 (58.1%)	15(71.4%)	10(45.5%)	0.084
Women (number; %)	18 (41.9%)	6 (28.6%)	12 (54.5%)	
Dry eye diagnosis^a^	9:29:5	3:14:4	6:15:1	0.245

All values are expressed as means ± standard deviation (SD).

^a^Data are presented as the following ratio: confirmed dry eye:probable dry eye:no dry eye

#### Effects of Royal Jelly on objective dry eye parameters

**Fig 2:**
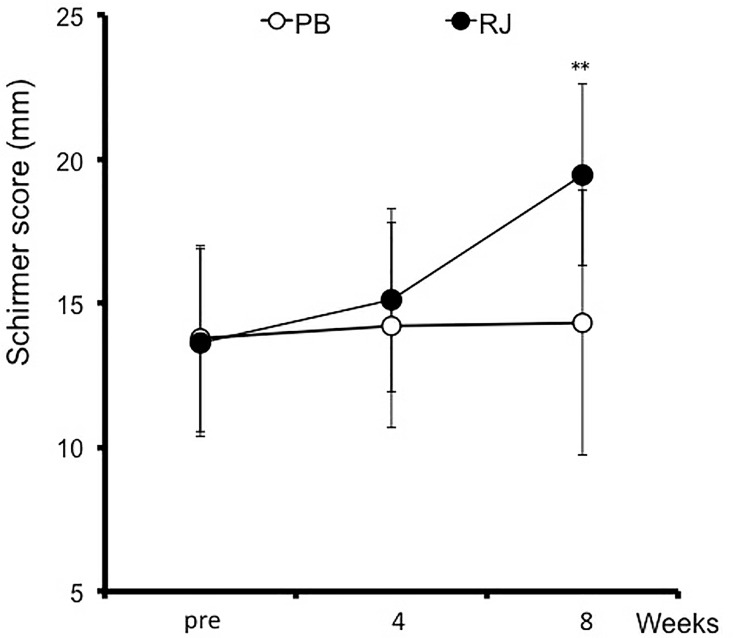
Change in Schirmer values in patients with dry eye symptoms who received royal jelly (RJ) or placebo (PB) tablets. Error bars represent standard deviation. **p<0.01 vs. pre-treatment values.

**Fig 3:**
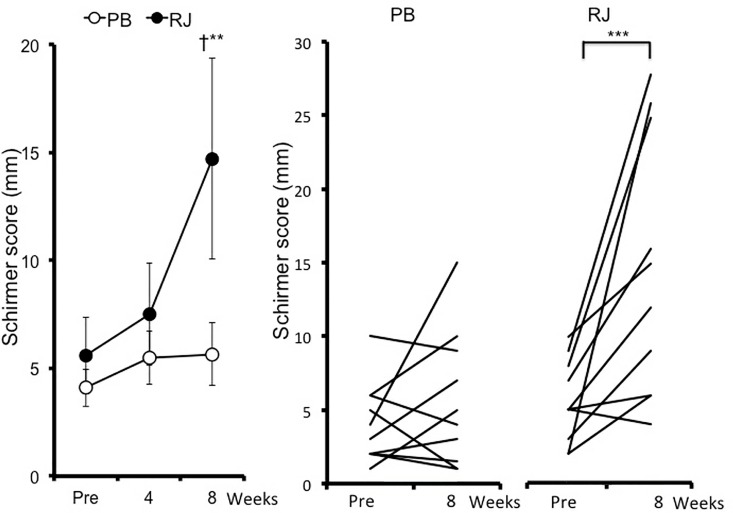
Change in Schirmer (baseline, ≤10 mm) values in patients with dry eye symptoms who received royal jelly (RJ) or placebo (PB) tablet. Data represent mean ± standard deviation **p<0.01, ***p<0.005 vs. pre-treatment values Between-group score comparisons at each time point (baseline, week 4, and week 8) were performed using the Aspin-Welch test (†*p*<0.05).

Objective dry eye parameters are summarized in Table 2. BUT was significantly higher at week 4 and 8 than at baseline in the RJ group (*p* = 0.0324 and *p* = 0.0396 respectively; Table 2). BUT at week 4 in the RJ group was significantly higher than that of placebo (*p* = 0.0271; Table 2). The fluorescein staining score have no significant differences between baseline and each point, and there was no significant difference between the two groups (Table 2). Although the Schirmer value increased at week 8 from baseline (*p* = 0.0009; Fig 2), there was no significant difference between the two groups. Patients with a baseline Schirmer value of ≤10 (n = 10) in the RJ group showed a significant increase at week 8 (*p* = 0.0005; Fig 3, Table 2) and a significant increase compared with those in the placebo group (*p* = 0.0051; Fig 3, Table 2). Meibum grades showed no significant differences between groups (Table 2). The DEQS total score was significantly lower at weeks 4 and 8 than at baseline in the placebo group (*p* = 0.0229 at week 4, *p* <0.0001 at week 8; Table 2). However, there were no significant differences between the two groups. The FVAΔshowed no significant differences between groups (Table 2).

**Table 2:** Effects of royal jelly (RJ) on dry eye symptoms assessed by ophthalmological examinations.

		RJ group		Placebo group		*p*^b^
		Mean ± SD	*p*^a^	Mean ± SD	*p*^a^	
BUT	Baseline	4.53 ± 3.17	-	3.76 ± 2.51	-	0.9653
	4 weeks	6.42 ± 3.44	**0.0324**	3.44 ± 2.10	0.9932	**0.0271**
	8 weeks	6.21 ± 2.90	**0.0396**	5.27 ± 2.55	0.3519	0.7668
Fluorescein staining score	Baseline	1.58 ± 1.61	-	1.64 ± 0.93	-	0.9902
	4 weeks	1.44 ± 1.35	0.9786	1.24 ± 1.35	0.2159	0.9991
	8 weeks	1.17 ± 0.90	0.8736	1.29 ± 0.99	0.3164	1
Schirmer test (mm)	Baseline	13.63 ± 10.63	-	13.76 ± 13.77	-	1
	4 weeks	15.11 ± 11.40	0.8897	14.24 ± 13.44	0.9995	0.9999
	8 weeks	19.47 ± 11.66	**0.0009**	14.32 ± 13.36	0.9988	0.8109
Schirmer test (≤10 mm)	Baseline	5.60 ± 2.84	-	4.10 ± 2.73	-	0.9878
	4 weeks	7.50 ± 6.02	0.9221	5.50 ± 3.92	0.9782	0.9572
	8 weeks	14.70 ± 8.93	**0.0005**	5.56 ± 4.59	0.9661	**0.0051**
Meibum grade	Baseline	0.47 ± 0.61	-	0.77 ±1.03	-	0.9044
	4 weeks	0.39 ± 0.50	0.982	0.63 ± 1.03	0.9898	0.9044
	8 weeks	0.63 ± 0.76	0.9098	0.77 ± 1.03	1	0.9969
DEQS	Baseline	28.23 ± 19.60	-	32.86 ±16.50	-	0.9848
	4 weeks	21.05 ± 16.05	0.1639	21.54 ± 14.34	**0.0229**	1
	8 weeks	21.67 ± 21.69	0.2451	15.52 ± 13.09	**<0.0001**	0.8497
FVAΔ	Baseline	0	-	0	-	-
	4 weeks	0.10 ± 0.14	0.4822	0.03 ± 0.22	0.9939	0.9076
	8 weeks	0.09 ± 0.33	0.5820	0.03 ± 0.23	0.9966	0.9311

BUT, tear film break-up time; DEQS, Dry Eye-Related Quality-of-Life Score; FVA, functional visual acuity; SD, standard deviation.

All values are expressed as means ± SD.

Dry eye symptoms were analyzed by repeated measures analysis of variance followed by Tukey's honest significant difference post hoc test.

^a^Within-group score comparisons between baseline and each time point (week4, week 8) were performed using paired *t*-tests.

^b^Between-group score comparisons at each time point (baseline, week4, week 8) were assessed using the Aspin–Welch test or Student’s *t*-test after the F-test.

#### Safety and tolerability

Table 3 summarizes the results of biochemical analyses. None of the 37 patients (0.0%) reported any adverse events or side effects.

**Table 3:** Biochemical analyses for patients with dry eye symptoms who received royal jelly (RJ) or placebo (PB) tablets.

	Treatment	Baseline	8 weeks	*p*-values		
				Baseline vs. 8 wks	PB vs. RJ	
					Baseline	8 wks
Total protein	PB	7.34 ± 0.40	7.25 ± 0.42	0.43	0.66	1.00
	RJ	7.39 ± 0.34	7.25 ± 0.42	**0.05**		
GOT	PB	18.06 ± 4.10	19.18 ± 6.82	0.38	0.40	0.49
	RJ	21.53 ± 16.86	21.8 ± 14.99	0.73		
GPT	PB	16.12 ± 7.11	17.67 ± 9.26	0.21	0.25	0.28
	RJ	27.68 ± 41.50	27.2 ± 36.02	0.78		
LDH	PB	182.59 ± 23.07	190.59 ± 56.39	0.55	0.77	0.36
	RJ	179.63 ± 34.60	176.16 ± 30.20	0.17		
ALP	PB	182.35 ± 59.07	175.24 ± 55.79	0.52	0.44	0.20
	RJ	196.32 ± 47.49	199.74 ± 55.72	0.44		
γ-GTP	PB	21.35 ± 8.87	21.29 ± 10.12	0.94	0.65	0.75
	RJ	23.74 ± 20.85	22.90 ± 18.81	0.31		
UN	PB	12.59 ± 2.80	11.26 ± 2.70	**0.005**	0.50	0.38
	RJ	12.05 ± 1.94	12.13 ± 3.13	0.92		
Creatine	PB	0.77 ± 0.18	0.72 ± 0.18	-*0.004	0.86	0.87
	RJ	0.78 ± 0.13	0.73 ± 0.10	**0.003**		
Uric acid	PB	5.15 ± 1.25	5.28 ± 1.39	0.40	0.24	0.36
	RJ	5.68 ± 1.39	5.71 ± 1.36	0.86		
Total cholesterol	PB	200.29 ± 32.96	199.47± 36.39	0.89	0.32	0.18
	RJ	187.80 ± 40.18	182.90 ± 36.90	0.20		
LDL-C	PB	116.65 ± 33.23	115.41 ± 31.07	0.83	0.69	0.47
	RJ	111.95 ± 37.36	107.53 ± 33.72	0.15		
HDL-C	PB	66.82 ± 19.50	64.82 ± 19.50	0.42	0.15	0.17
	RJ	59.42 ± 9.97	57.16 ± 13.22	0.15		
Triglyceride	PB	104.90 ± 43.61	101.53 ± 55.80	0.76	0.69	0.74
	RJ	98.63 ± 48.76	119.68 ± 87.13	0.53		
Glucose	PB	82.59 ± 8.70	88.00 ± 1402	0.24	0.24	0.43
	RJ	87.32 ± 14.62	91.90 ± 14.91	0.31		
HbA1c	PB	5.15 ± 0.17	5.17 ± 0.19	0.33	**0.02**	**0.02**
	RJ	5.33 ± 0.25	5.34 ± 0.23	0.73		

AST, aspartate aminotransferase; ALT, alanine transaminase; LDH, lactate dehydrogenase; ALP, alkaline phosphatase; γ-GTP, gamma-glutamyl transferase; UN, urea nitrogen; Chol, cholesterol; LDL-C, low-density lipoprotein cholesterol; HDL-C, high-density lipoprotein cholesterol, HbA1c; hemoglobin A1c

All data are expressed as mean ± standard deviation.

### Animal study results

**Fig 4:**
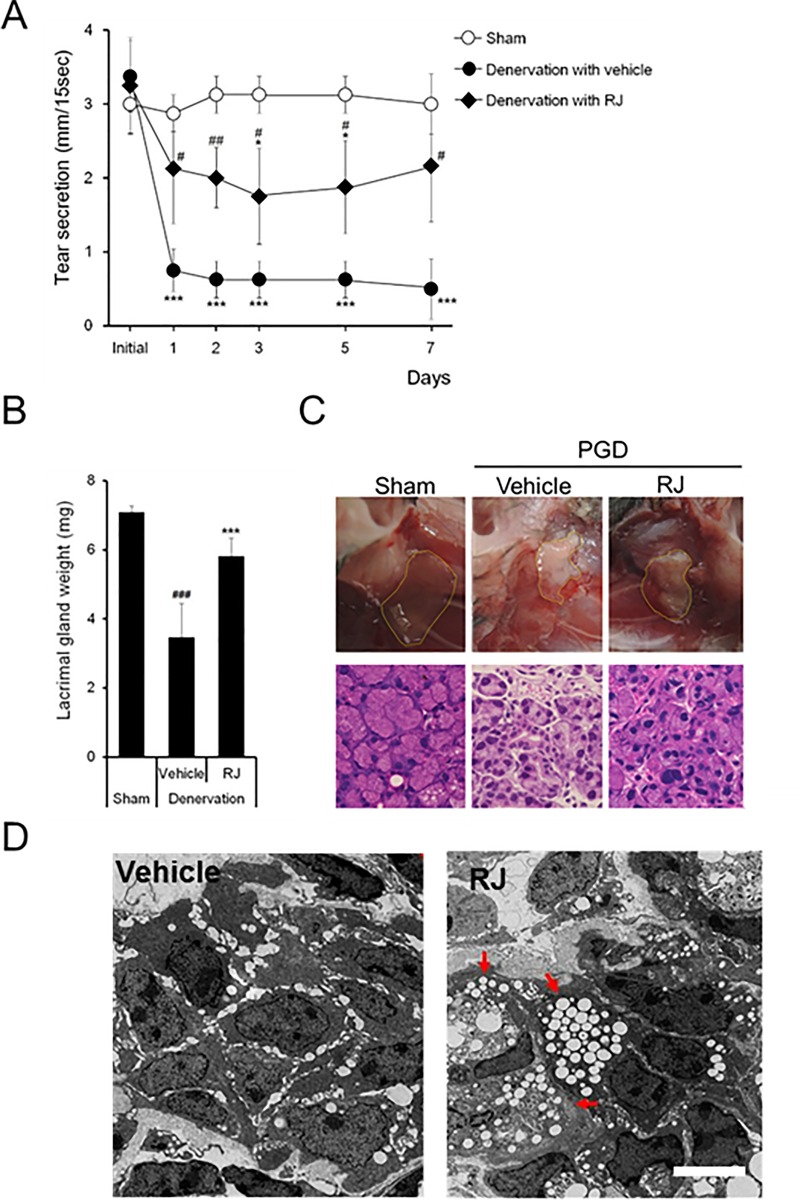
Direct maintenance of acinar cell activity in lacrimal glands (LGs) by orally administrated royal jelly. A: Effects of RJ on decreased tear secretion after postganglionic denervation (PGD; six mice). B: LG weight (n = 4–5 lacrimal glands). C: Histopathological changes in LGs Gross appearance of LG (upper) and hematoxylin and eosin staining of LGs (lower). The scale bar is 3 mm (upper) and 50 μm (lower). E: Typical transmission electron microscopy images of an acinar cell after vehicle (left) and RJ (right) treatment. The arrow indicates vesicle-rich acinar cells. The scale bar is 5 µm. All data represent mean ± standard deviation. *p<0.05, **p<0.01, ***p<0.001 versus sham. #<0.05, ## *p*<0.01 versus denervation with vehicle

We investigated the mechanisms underlying the increased Schirmer values, which determine tear secretion, in the RJ group using a mouse PGD dry eye model. Fig 4A shows the effect of RJ oral administration on tear secretion reduction after PGD. Tear secretion remained unchanged after the sham operation. In the denervation with vehicle group, tear secretion decreased by approximately 80% on day 1 compared with before PGD, and this decrease was sustained from day 1 to day 7. Significant differences in denervation with the vehicle group were observed from day 1 to day 7, compared with the sham operation. This suggests that an interruption of neuronal stimuli from the central nervous system to the LGs was achieved by PGD (*p* = 0.00013).

Orally repeated administration of RJ suppressed the reduction in tear secretion by PGD. The values were approximately half those of the sham operation. Significant differences were observed on day 3 (*p* = 0.017) and 5 (*p* = 0.021), and from day 1 to day 7 (*p* = 0.026 at day 1, *p* = 0.009 at day 2, *p* = 0.032 at day3, *p =* 0.021 at day 5, and *p* = 0.015 at day 7) compared with the sham and denervation with vehicle groups, respectively. LG weight significantly decreased in the vehicle group compared with that in the sham group. RJ treatment significantly suppressed the reduction in LG weight observed in the vehicle group (Fig 4B). Typical macroscopic examinations of LGs are shown in Fig 4C (upper). Histopathological evaluation of LGs showed that a decrease in the size of acinar cells after PGD was maintained in the RJ group (Fig 4C; lower). TEM analysis of LG acinar cells showed an absence of secretory vesicles in the vehicle group, while in the RJ group, secretory vesicles filled the acinar cells (Fig 4D).

## Discussion

In the present study, we found that oral RJ supplementation for 8 weeks can improve tear secretion, as assessed by Schirmer score, in patients with dry eye symptoms. In addition, we showed potential involvement of RJ-induced restoration of LG function in this effect. We also observed that BUT increased after 4 weeks of daily oral RJ supplementation. With regard to tear production, the increase tended to be higher in the RJ group than in the placebo. In particular, patients with a baseline Schirmer value of ≤10 mm showed a significant increase compared with values at baseline and those in the placebo group.

Dry eye is associated with oxidative stress [24–27]. To date, a number of studies related to dietary foods for dry eye have reported that the effects of these foods may be related to anti-inflammatory or anti-oxidative stress [24,28,29]. RJ has also been reported to exhibit a novel function of tear secretion from LGs, in addition to anti-inflammatory or anti-oxidative effects [16]. Our previous study demonstrated that oral administration of RJ enhanced tear secretion capacity in blink-suppressed dry eye models. These effects occurred alongside an increase in ATP, mitochondrial function, and phosphorylation of AMPK, reflecting the restoration of energy in LGs [16]. The present animal study further demonstrated that oral administration of RJ preserves tear secretion and LG structure under a condition whereby neuronal stimuli to LGs are interrupted. These findings correspond to the finding of increased tear production in the present study in humans and indicate that direct effects on LGs are a potential mechanism of action of dietary RJ supplements in patients with dry eye symptoms. Furthermore, we found that RJ improved tear stability as assessed by BUT evaluations.

This study has several limitations. First was the short study period, which may have been a potential reason for the lack of an improvement in dry eye symptoms. Second, the sample size may have been too small and the study period too short for several measures to reach statistical significance. Previous studies of RJ have adopted longer observation periods and larger sample sizes than our study; however, the optimal sample size and study period to determine the ocular surface improvements are unknown. Further studies with longer observation periods and larger sample sizes may be required. Third, the patients of this study had subjective symptoms. Considering that RJ promotes tear secretion from the LG, patients with aqueous-deficient dry eye might have been more appropriate to study.

Currently, an increase in exposure to visual display terminals such as smartphones and portable games, at all ages has become a strong risk factor for dry eye and is considered a cause of the increasing number of affected patients [6]. Regardless of subtypes such as evaporative dry eye and tear deficiency dry eye, the current treatment for dry eye includes supportive measures including the use of eye drops and punctal plugs. The results of our study suggest that RJ is a promising fundamental alternative for increasing tear secretion and protecting LG function. The specific molecule of RJ that affects tear secretion should be elucidated in future studies. In conclusion, oral RJ administration is a safe and effective intervention for increasing the tear volume in human patients with dry eye.

## Supporting Information

S1 Text CONSORT Checklist. (DOC)

S2 Text Protocol in Japanese. (DOCX)

S3 Text Protocol in English. (DOCX)
