# Supplementary material for: Clinical Evaluation of a Royal Jelly Supplementation for the Restoration of Dry Eye: A Prospective Randomized Double Blind Placebo Controlled Study and an Experimental Mouse Model
Source: PLoS One. 2017 Jan 6;12(1):e0169069. doi: 10.1371/journal.pone.0169069 (PMC5217957; doi:10.1371/journal.pone.0169069)
Supplement: S2 Text — (DOCX) [file pone.0169069.s002.docx]

試験実施計画書

試験名：ロイヤルゼリー含有食品摂取によるドライアイ改善効果確認試験

試験実施計画書番号：

作成年月日： 2014年 03月 30日版数：第１版

**1．**試験計画の概要

(1)試験食品：ロイヤルゼリー含有食品（被験食品）、ロイヤルゼリー非含有食品（プラセボ食品）

(2)目的：ロイヤルゼリー含有食品の 8週間連続摂取時の眼科検査項目並びに、ドライアイに関する主観的評価に及ぼす影響を、プラセボ食品を対照として検討する。

(3)対象： ドライアイ自覚症状のある 20歳から 60歳の男女

(4)摂取開始被験者数： 50名（被験食品 25名、プラセボ食品 25名）

(5)試験デザイン：無作為化プラセボ対照二重盲検並行群間比較試験

(6)関与成分の摂取量：ロイヤルゼリー 7200mg (生換算) /日

(7)摂取方法： 1日に 3回、カプセル 2粒を毎食後に噛まずに十分な量の水と共に摂取する。

(8)摂取期間： 8週間

(9)試験の実施予定期間： 2014年 5月から 2015年 3月まで

**2.**試験の目的

ドライアイ自覚症状がある 20歳から 60歳の男女にロイヤルゼリー含有食品を 8週間連続摂取させた際の、眼科検査項目並びに、ドライアイに関する主観的評価に及ぼす影響を、プラセボ食品を対照として検討する。

**3.**試験実施体制

3.1.試験監修医師

氏名：坪田一男

　 所属・役職：慶應義塾大学医学部眼科学教室・教授

主な役割：試験実施計画への助言及び確認、試験実施結果の助言及び確認を行う。

3.2.試験責任医師、試験分担医師

(1)試験責任医師

氏名：井上佐智子

所属：羽根木の森アイクリニック　院長

主な役割：試験に係る業務を統括し、管理責任を負い、被験者への指示及び説明、 被験者の同意の取得、問診及び有害事象の確認と判定、ケースリポートフォームの 作成、検査実施体制の管理を行う。また有害事象について、必要に応じて処置を行 う。

(2)試験分担医師

氏名：川島素子

所属：慶應義塾大学医学部眼科学教室

主な役割：試験責任医師をサポートし、被験者への指示及び説明、問診及び有害事 象の確認と必要に応じた処置を行う。

(3)試験分担研究者

氏名：中村滋

所属：慶應義塾大学医学部眼科学教室

主な役割：試験責任医師・分担医師をサポートし、試験実施計画への助言、実施結 果の検討および助言を行う。

3.3.試験実施医療機関

名称：羽根木の森アイクリニック

所在地：〒155−0033　東京都世田谷区代田4-26-4

主な役割：試験を行う医療機関であり、身体所見の判定並びに理学検査、眼科検査 を行う。また被験者の管理、試験実施体制の整備を行う。

3.4.倫理審査委員会

名称：白澤治験センター倫理審査委員会

委員長：安楽岡滋先生

所在地：〒 群馬県館林市

役割：試験実施計画及び試験食品に関する資料に基づき、疫学指針及び倫理的観点、科学的観点から審査し、文書により意見を述べる。

4.試験食品

4.1.試験食品の種類及び名称

(1)被験食品：酵素分解ローヤルゼリー（以下RJ）含有食品

(2)プラセボ食品：RJ非含有食品

4.2.被験食品の食経験

株式会社山田養蜂場が製造・販売している酵素分解ローヤルゼリー含有健康補助食品は 2008年発売から 2013年までの販売実績が100万本以上、販売実績があり、10万人以上がすでに摂取している。これまでこれらの食品摂取によって、健康に重篤な影響を及ぼしたという報告はされていない。

4.3.関与成分：ローヤルゼリー

4.4.試験食品の組成等

| 1粒あたり（概算値） | | 被験食品※ | プラセボ食品 |
| --- | --- | --- | --- |
| エネルギー | （kcal） | 2.2 | 2.03 |
| たんぱく質 | （g） | 0.16 | 0.15x10-3 |
| 脂質 | （g） | 0.03 | 0.025 |
| 炭水化物 | （g） | 0.31 | 0.43 |
| ナトリウム | （mg） | 9.43 | 0.045 |

　　　※1粒あたり、酵素分解RJ 1200mg (生換算)含有

4.5.試験食品の摂取量、摂取方法、摂取期間及び関与成分摂取量の設定

(1)試験食品の摂取量、摂取方法

1日 3回、2粒を毎食後に噛まずに十分な水と共に摂取する。食後に摂取し忘れた場合は、当日中に 計6粒を摂取し、数日分まとめての摂取は禁止とする。当日中に、摂取した時刻と量を日誌に記録し、摂取し忘れた場合も当日の日誌に記載するよう指導する。検査日当日は摂取せずに来院し、 4週目の検査日は検査終了後に摂取する。

(2)試験食品の摂取期間 8週間（56日間）とする。

(3)関与成分摂取量の設定根拠

RJ含有食品の 1日摂取目安は 1日 3-6粒（1粒あたり、酵素分解RJ 1200mg (生換算)含有）だが、動物実験で300mg/dayで効果が明確であったため、本試験の 1日摂取量を 6粒（RJ　7200mg）に設定した。また、既に多数販売されているRJ関連食品の試験で、安全性が確認されている摂取量をもとに 1日の摂取量を設定した。

**5.**試験デザイン無作為化プラセボ対照二重盲検並行群間比較試験

**6.**被験者数及び割付方法

6.1.被験者数

摂取開始被験者数は 50名とし、その内訳は被験食品摂取開始者 25名、プラセボ食品摂取開始者 25名とする。

6.2.割付方法

割付担当者は乱数を用いて割付表を作成し、試験食品に割付番号を付与する。割付表は割付担当者が封緘し、割付表開封時まで密封保管する。解析対象者及びデータ固定後、割付担当者は割付表を開封し情報を開示する。ただし重篤な有害事象が発生し、緊急を要する場合には、必要に応じて割付表を開封し、必要最低限の情報のみを開示する。

7.被験者の選択基準及び除外基準

次の選択基準に合致し、除外基準に抵触しない者を被験者として選択する。

7.1.選択基準

(1)年齢 20歳以上 60歳以下の男女

(2) ドライアイの自覚のある者

7.2.除外基準

(1)事前検査時の視力検査において、視力矯正具を装用した際の視力が 1.0未満と判断される者

(2)強度近視（-6D以上）の者

(3)本試験食品の有効成分（RJ）を強化している、あるいは視覚機能改善効果作用を有する健康食品や医薬部外品、一般医薬品（点眼薬含む）を常用している者

(4)現在、眼科疾患がある者、又は重篤な眼科疾患の既往歴がある者

(5)以下の視力障害と診断されたことがある者白内障（先天性、老人性、糖尿病性、併発性、外傷性）緑内障（慢性、急性、先天性、続発性）

(6) 7月から 12月までの期間で花粉症を発症する恐れのある者、喘息の持病のある者

(7)服薬治療中の者、又は投薬治療を必要とした重篤な疾患既往歴を有する者

(8)レーシック施術後、 3ヵ月以内の者

(9)試験食品によりアレルギーを引き起こす恐れのある者

(10)本試験への参加同意取得前 1ヵ月以内に他の臨床試験に参加していた者、あるいは本試験の参加同意取得後に他の臨床試験に参加する予定のある者

(11)試験期間中に妊娠、授乳の予定がある者

(12)その他、試験責任医師が被験者として不適当と判断した者

**8.**被験者の同意

8.1.同意取得時期 1回目の検査実施前に同意を文書で取得する（検査スケジュールは後述項参照）。

8.2.説明文書の内容被験者に対する説明の内容は、以下の事項を含むものとする。

・ 当医療機関、試験責任医師等の氏名

・ 当該試験（研究）の目的、意義及び方法、期間

・ 当該試験（研究）への参加が任意であること

・ 当該試験（研究）の実施に同意しない場合であっても何ら不利益を受けることはないこと

・ 被験者が当該試験（研究）に同意した場合であっても随時これを撤回できること

・ 当該試験（研究）に参加することにより期待される利益及び起こりうる危険並びに必然的に伴う不快な状態

・ 危険又は必然的に伴う不快な状態が起こりうる場合の、当該試験（研究）に伴う補償等の対応

・ 当該試験（研究）に係る資金源、起こりうる利害の衝突及び研究者等の関連組織との関わり(利益相反)

・ 個人情報等の取扱い

・ 被験者を特定できないようにした上で、試験（研究）の成果が公表される可能性があること

・ 共同試験（研究）を行う場合は、共同試験（研究）であることなど

・ 個人情報等の取扱に関する苦情の申出先

・ 試験（研究）参加に当って守っていただきたいこと

・ 新しい情報提供及び試験計画の変更について

・ 試験（研究）費用の負担、協力費について

・ 試験（研究）終了後の資料の保存、利用又は廃棄の方法（他の試験研究への利用の可能性と予測される研究内容を含む）

・ 倫理審査委員会とその議事要旨の公開について

8.3.同意取得方法

ヘルシンキ宣言の精神に則り、被験者が試験に参加する前（ 1回目の検査実施前）に、倫理審査委員会により承認された最新の説明文書・同意文書を手渡した上で十分な説明を行い,被験者の質問に対して被験者が満足できるよう回答に努める。その際、被験者に質問する機会と試験参加の判断を行うに十分な時間を与える。試験責任（分担）医師は被験者の自由意思による同意を文書で得る。同意文書には試験責任（分担）医師及び被験者が署名若しくは記名捺印し、日付を記入する。

8.4.被験者の意思に影響を与える情報の提供並びに説明文書及び同意文書の改訂試験依頼者は、被験者の同意に関連し得る新たな重要な情報が得られた場合、速やかに試験責任医師に文書で通知する。試験責任（分担）医師は、被験者の同意に関連し得る新たな重要な情報が得られた場合、速やかに被験者に説明し、試験継続の意思の確認を行う。試験責任医師は入手した情報に基づき説明文書及び同意文書を改訂する必要があると認めた場合は速やかに改訂し、倫理審査委員会の承認を得たうえで速やかに試験参加中の被験者に再度同意取得（試験継続の意思の確認）を行う。

**9.**個人情報等の保護

本試験に関与する者は、同意文書、ケースリポートフォームの取扱い及びデータの公表に際しては、被験者の個人情報並びにプライバシーの保護について十分配慮するものとする。本試験はケースリポートフォームの作成、論文の公表、規制当局から求められた場合の資料提出等においては、被験者を特定できない方策（被験者の表記に関して被験者識別番号で行う等）を講じ、被験者の秘密を保全する。

| 検査日 | | 事前期間 |  | 摂取期間（ 8週間） | | |
| --- | --- | --- | --- | --- | --- | --- |
|  | | 事前 |  | 摂取前 | 摂取 4週目 | 摂取 8週目 |
| 選択・割付 | |  | ○ |  |  |  |
| 生活習慣調査票 | | ○ |  |  |  |  |
| 問診 | | ○ |  | ○ | ○ | ○ |
| 身体測定 | 身長 | ○ |  |  |  |  |
|  | 体重 | ○ |  | ○ | ○ | ○ |
| 理学検査 | 収縮期血圧 | ○ |  | ○ | ○ | ○ |
|  | 拡張期血圧 | ○ |  | ○ | ○ | ○ |
|  | 脈拍数 | ○ |  | ○ | ○ | ○ |
|  | 血液生化学検査 |  |  | ○ |  | ○ |
|  | 眼底検査 | ○ |  |  |  |  |
|  | 視力・屈折検査 | ○ |  |  |  |  |
|  | 実用視力 |  |  | ○ | ○ | ○ |
|  | BUT | ○ |  | ○ | ○ | ○ |
|  | 角結膜障害 | ○ |  | ○ | ○ | ○ |
|  | シルマーテスト | ○ |  | ○ | ○ | ○ |
| ドライアイ主観的調査 | DEQS | ○ |  | ○ | ○ | ○ |
| 試験食品の摂取 | |  |  | 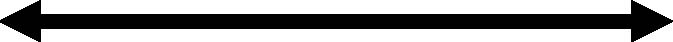 | | |
| 日誌の記録 | |  |  | 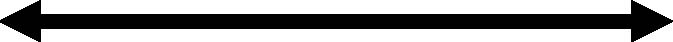 | | |

**10.**スケジュール及び検査内容

10.1.試験スケジュール

(1)同意を取得した被験者候補に事前検査を行い、生活習慣アンケート、問診、身体測定、理学検査、眼科検査（眼底検査、視力、屈折率、シルマー試験）及び、ドライアイに関する主観的評価（アンケート）を実施する。

(2)事前検査の結果から試験の目的に適した被験者を 50名選択する。

(3)組み入れた被験者を摂取前検査のために来院させ、問診、身体測定、理学検査、眼科検査（実用視力、BUT（break up time of tear film：涙液層破壊時間）、角結膜障害）及びドライアイに関する主観的評価（アンケート）を実施する。

(4)摂取前検査終了後に、組み入れた被験者に割り付けられた試験食品を配布し、翌日より試験食品の摂取を開始させる。また、試験食品を摂取した時間等を記載する日誌の記録を開始させる。

(5)摂取 4週目、及び 8週目の各来院時には問診、身体測定、理学検査、眼科検査（実用視力、BUT、角結膜障害、シルマー試験）を実施する。

10.2.検査日

計 4回の来院時に検査を行う。被験者の都合等で規定の検査日に検査ができない場合は、責任医師あるいは分担医師の判断により、規定の検査日の前後 7日間の範囲で検査日を変更することができる。また、必要に応じて、再検査及び追跡検査を行うことができる。

10.3.調査及び検査の内容

前頁スケジュールを参照。

| 項目 | 内容 | |
| --- | --- | --- |
| 生活習慣アンケート | 既往歴、医薬品や健康食品の摂取、アレルギーの有無、喫煙の状況、飲酒傾向、ドライアイの自覚症状（DEQS）など | |
| 問診 | 体調の確認、有害事象の有無の確認 | |
| 身体測定 | 身長*1、体重、BMI | |
| 理学検査 | 収縮期血圧、拡張期血圧、脈拍数, 血液生化学検査[総蛋白、AST(GOT)、ALT(GPT)、γ-GTP、ｱﾙｶﾘﾌｫｽﾌｧﾀ-ｾﾞ(ALP)、LD(LDH)、総ｺﾚｽﾃﾛ-ﾙ(T-Cho)、HDL-ｺﾚｽﾃﾛ-ﾙ、LDL-ｺﾚｽﾃﾛ-ﾙ、中性脂肪(TG)、尿素窒素(UN)、ｸﾚｱﾁﾝ、尿酸(UA)、ｸﾞﾙｺ-ｽ、HbA1c(NGSP)] | |
|  | 眼底検査 | 問診時に医師による診察 *1 |
|  | 視力 | 視覚検査装置による測定 *1 |
|  | 実用視力・コントラスト視力 | 実用視力計による検査 *2 |
|  | 屈折率 | オートレフ・ケラトメーターによる検査 *1 |
|  | BUT | フルオレセイン BUT*2 |
|  | 角結膜障害 | フルオレセイン染色試験 *2 |
|  | シルマー試験 | *3 |

1. 1：事前検査のみ実施
2. 2：摂取前検査から実施
3. 3：事前検査および 8週目検査時に実施

10.4.被験者の制限事項（注意事項）

(1)試験食品の摂取期間中は、毎日、毎食後、試験食品 2粒を摂取させ、日誌を記録させる。

(2)試験実施期間中は、試験参加前からの食事・飲酒・運動・睡眠等の生活習慣を維持し、日常範囲を大きく逸脱する過度な運動、節食や過食を避ける。

(3)試験実施期間中は、新たに健康食品やサプリメントを摂取しない。

(4)検査日の前日から当日の検査終了までは、禁酒とする。

(5)検査日の前日から当日の検査終了までコンタクトレンズの装用を禁止する。当日は事前検査と同じ眼鏡を持参させる。

(6)検査当日は来院 2時間前までに指定した食事を終了させる（水の摂取は可）。

(7)検査当日は来院 2時間以上前から禁煙させる。

**11.**予想される副次作用現段階で予想される副次作用はないが、食品の一般的な症状として、ごくまれに腹痛や下痢などの腹部症状が現れる可能性が考えられる。

**12.**有害事象

12.1.有害事象の定義試験食品摂取後に被験者に新たに発現した、あるいは悪化したあらゆる好ましくない医療上のできごと（試験食品摂取後に発現した自覚症状、他覚所見、検査値の異常変動）を有害事象とし、試験食品との因果関係の有無にかかわらない。

12.2.有害事象の判断被験者の自覚症状、他覚所見としての有害事象は、試験責任医師が判断する。被験者個々の検査値の異常変動（有害事象）については、試験実施医療機関で定める基準値を基にして、NCI、CTC及び日本化学療法学会が定める異常変動の判定基準（Chemothrapy.2010.58（4
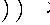
を参考として、試験責任医師が有害事象の判断をする。

12.3.有害事象の収集・記録

有害事象が発現した場合は、必要に応じて適切な処置を行うとともに、その症状・所見の内容（診断名）、有害事象の程度、重篤か否か、試験食品との関連性、発現日、消失日、本試験が継続できるか否か、病院での処置の有無（処置の内容）、転帰及び医師のコメント等についてケースリポートフォームに記載する。発現日とは、有害事象が発現した日（特定できない場合は確認した日）とし、消失日は有害事象が消失した日（特定できない場合は確認した日、あるいは消失が認められない場合は転帰を確認した日）とする。

なお、有害事象の評価は以下の基準で判定する。

(1)程度

- - 1. 軽 度：日常生活に特に支障を来さない
    2. 中等度：日常生活に支障を来す
    3. 高 度：日常生活がほとんど送れない

(2)重篤発現した有害事象が以下に該当する場合は、重篤な有害事象とする。

- - 1. 死亡
    2. 死亡につながるおそれのある事象（生命を脅かす事象）
    3. 治療のために入院又は入院期間の延長が必要とされる事象
    4. 障害（日常生活に支障を来たす程度の機能不全を示すもの）
    5. 障害につながるおそれのある事象
    6. 上記にあげた事象（障害）に準じて重篤である事象
    7. 後世代における先天性の疾病又は異常

(3)試験食品との関連性

- - 1. なし：明らかに試験食品以外の原因が特定できる
    2. 多分なし：試験食品摂取を原因とすることに、合理的な時間的関連やその他の医学 的理由が見出されない
    3. あるかもしれない：試験食品摂取から合理的な時間的関連や、その他の医学的理由 が認められるが、試験食品以外の原因も疑われる
    4. あり：試験食品摂取から合理的な時間的関連や、その他の医学的理由が認められ､ 試験食品以外の原因が疑われない

(4)転帰

- - 1. 回復・消失：症状、所見が消失、検査値が正常化又は摂取前値へ回復
    2. 軽快：程度が 1段階以上改善、軽度の症状については症状又は所見がほぼ消失、検 査値が正常域付近又は摂取前値付近へ回復
    3. 不変：症状、所見、検査値がほとんど変化なし
    4. 悪化：症状、所見、検査値の増悪が認められる
    5. 不明：症状・異常値の追跡に手を尽くしたが、経過を追うことができなかった（「死 亡」も含む）（例：転居など）

(5)試験食品摂取の継続性有害事象が発現した際に、試験参加継続に関する措置を以下の基準で判定する。

- - 1. 継続：試験食品の摂取を継続する（試験を継続する）
    2. 中断：一時的に試験食品の摂取を中断し経過をみる
    3. 中止：試験食品の摂取を中止する

12.4.副次作用有害事象のうち、試験食品との関連性が「あるかもしれない」及び「ある」と判定された試験食品との因果関係を完全に否定できない事象を指す。

12.5.追跡調査有害事象が認められた場合は、その事象が消失もしくは回復傾向が認められるまで、あるいは試験責任医師が実施の必要がないと判断するまで、追跡調査を行う。

12.6.重篤な有害事象発現時の報告手順

観察された有害事象が重篤である場合、試験責任医師は発現を知った後、速やかに試験実施医療機関の長に報告し、重篤な有害事象に関する報告書を作成し、試験実施医療機関の長に提出する。

**13.**中止と脱落

13.1.定義中止とは、「本試験を完了する前に、試験責任医師の判断で試験を打ち切る場合」とする。脱落とは、「本試験を完了する前に、被験者の都合や意思により試験が打ち切られた場合」とする。

13.2.中止

試験責任医師は次の項目に該当する事象が生じた場合は、当該被験者又は本試験全体を中止できるものとする。

(1)当該被験者における中止

被験者の安全性を損なう恐れがあると判断した場合

重篤な臨床的異常の発現又は事故が発生し、本試験の継続が困難な場合

被験者による、試験実施計画書からの重大な又は継続した不遵守が判明した場合

その他、試験責任医師が試験中止を適切と判断した場合

(2)本試験の中止

試験期間中に、試験依頼者に試験全体を中止せざるを得ない理由が生じた場合、試験依頼者は開発業務受託機関と協議し、試験の中止及び中止後の方策を決定する。試験依頼者は、速やかに試験中止及びその理由を試験責任医師、倫理審査委員会、及び試験実施医療機関に文書により通知するものとする。また、試験責任医師は試験全体の中止の通知を受けた場合、及び被験者の本試験への参加の継続に影響を与えるような情報等を入手した場合には、被験者にこれらの情報を提供するものとする。

倫理審査委員会により、中止後の対応において何らかの要求があった場合、試験依頼者は、開発業務受託機関及び試験責任医師と協議し、対応する。

13.3.脱落被験者が本試験に参加することに同意した後に、被験者の都合や意思により脱落する場合は、試験責任医師は当該被験者の試験を中止する。

13.4.中止（脱落）時の対応中止（脱落）の場合は、中止（脱落）日及び理由をケースリポートフォームに記載する。

**14.**解析対象者の選択

14.1.有効性解析対象者

解析対象者は、所定の試験スケジュールや試験内容をすべて終了した被験者のうち、以下の基準に該当する被験者を除外した者とする。

【解析対象除外基準】

(1)試験食品の摂取率が 80%を下回った者

(2)日誌記録の欠損など、検査結果の信頼性を損なう行為が顕著に見られる者

(3)除外基準に該当していたことが試験組み入れ後に明らかになった者や、試験期間中に制限事項（注意事項）を遵守できないことが判明した者。

(4)その他、除外することが適当と考えられる明らかな理由があった者

14.2.安全性解析対象者

(1)有害事象一度でも試験食品を摂取したことのある被験者を対象とする。

(2)検査値所定の試験スケジュールや試験内容を全て終了した被験者とする。

**15.**評価方法

151.有効性の評価

(1)主要評価項目：ドライアイ自覚症状、BUT、角結膜障害、シルマー値

(2)副次評価項目：実用視力

(3)有効性の評価方法

各評価項目について、被験食品摂取群の摂取前及び摂取後の各時点における値と、プラセボ食品摂取群のそれとを 2標本 t検定を用いて比較する。また、被験食品摂取群の摂取前と摂取後の各時点における変化量もプラセボ食品摂取群のそれとを 2標本 t検定を用いて比較する。また、各群で摂取後各時点の値を、 1標本 t検定を用いて摂取前のそれと比較する。

16.2.安全性の評価

(1)評価項目

　問診、日誌（自覚症状）を用いて確認した有害事象及び体重、理学検査値の異常変動検　査値は、体重、理学検査（収縮期血圧、拡張期血圧、脈拍数、血液生化学検査値項目の変化を確認する。

(2)評価方法

有害事象及び異常変動は、発現件数を集計し、一覧表を作成する。同一の有害事象が同一被験者に複数回発現した場合は、 1件として取り扱う。

検査値の変化は、摂取前と摂取後各時点の測定値を、 1標本 t 検定を用いて比較する

.数値の表示及び有意水準数値は平均値±標準偏差（又は標準誤差）で示し、検定の有意水準は両側 5%とする。

16.3試験実施計画書の遵守、変更、逸脱、及び改訂

試験責任医師は、試験実施計画書を遵守し、試験依頼者との合意に基づく事前の文書による承認を得ることなく、試験実施計画書からの逸脱又は変更を行ってはならない。また、倫理審査委員会による承認の後に試験実施計画書の変更を行う場合は、改めて倫理審査委員会に変更事項の承認を得ることとする。

ただし、被験者の緊急の危険を回避する場合等、医療上やむを得ないものである場合、又は試験の事務的事項（例えば、電話番号の変更等）のみに関する変更である場合は、この限りではない。

**17.**ケースリポートフォームの作成試験責任医師は、各被験者の試験結果についてケースリポートフォームを作成し、記名捺印の上、試験依頼者に提出する。ケースリポートフォームのデータは原資料と矛盾してはならない。また、被験者の事前の承諾なしに個人情報を記載しないことを厳守する。

**18.**倫理

18.1.被験者の選定被験者となるべきものの選定にあたっては、人権保護の観点及び試験実施計画書の選択基準、除外基準に基づき、被験者の健康状態、年齢、性別、同意取得能力、試験責任医師との依存関係、他の試験への参加の有無等を考慮し、試験に参加を求めることの適否について慎重に検討する。同意の能力を欠く者については被験者としない。試験に参加しないことにより不当な不利益を受けるおそれのある者を選定する場合は、当該者の同意が自発的に行われるよう十分な配慮を行う。

18.2.ヘルシンキ宣言及び疫学研究に関する倫理指針本試験は、ヘルシンキ宣言の精神に則り、実施にあたっては疫学研究に関する倫理指針に従い実施する。

18.3.被験者に健康被害が生じた場合の対処

本試験に参加したことにより被験者に健康被害が生じた場合は、試験責任医師は速やかにその治療及びその他の必要な処置を講じる。ただし、被験者自身の過失又は故意によりその障害が発生した場合はこの限りでない。試験依頼者は当該責任を履行するために、保険その他の必要な措置を講じるものとする。

18.4.結果の説明

本試験に参加した被験者は、本試験終了後に、検査等の結果について試験責任医師あるいは試験分担医師から説明を受けることができる。

**19.**原資料等の直接閲覧の実施試験責任医師及び試験実施医療機関は、記録等の調査、確認のため、試験依頼者の担当者から要請があった場合は、調査又は原資料（同意文書、被験者より提出される日誌等の記録及びケースリポートフォームに記録されるデータの原本がある場合はその原本）の直接閲覧に応じるものとする。ただし、個人情報保護の観点から、個人情報を含む原資料等の直接閲覧には十分配慮をする。

**20.**金銭の支払い

21.1.本試験の費用本試験の費用については研究助成金を使用する(山田養蜂場みつばち研究助成基金)

21.2.試験協力費

被験者に支払われる試験協力費は、試験実施医療機関の規定に基づいて算定された額を支払う。

**21.**記録の保存

21．1.試験実施医療機関の長

　　試験実施医療機関の長は、試験実施医療機関において保存すべき文書、即ち原資料（同意文書、被験者より提出される日誌等の記録、及びケースリポートフォームに記録されるデータの原本がある場合はその原本）、契約書、倫理審査委員会審査資料に係わる書類、その他試験食品の管理記録等の試験に係わる業務の記録などを、試験の中止又は終了後 5年が経過する日まで保存する。ただし、試験依頼者がこれよりも長期間の保存を必要とする場合は、保存期間及び保存方法について協議を行う。また、実施医療機関に記録の保存に係わる規定がある場合には、当該規定に従う。

21.2.試験責任医師試験責任医師は、試験の実施に係わる保存すべき文書を試験実施医療機関の長の指示に従って保存する。

**22.**記録の廃棄

保存期間を経過した時点で、被験者の署名のある同意文書等の資料については、適切に廃棄（焼却等）する。匿名化された資料については、匿名化したまま適切に廃棄（焼却等）する。

**23.**試験実施予定期間

2014年 5月から 2015年 3月まで 以上
