# Supplementary material for: Clinical Evaluation of a Royal Jelly Supplementation for the Restoration of Dry Eye: A Prospective Randomized Double Blind Placebo Controlled Study and an Experimental Mouse Model
Source: PLoS One. 2017 Jan 6;12(1):e0169069. doi: 10.1371/journal.pone.0169069 (PMC5217957; doi:10.1371/journal.pone.0169069)
Supplement: S3 Text — (DOCX) [file pone.0169069.s003.docx]

Trial Protocol

Trial name: Investigation of the effect of consuming a food product containing royal jelly on dry eyes

Trial protocol number:

Date created: March 30, 2014

Edition number: First edition

**1. Trial protocol outline**

(1) Trial food: Food product containing royal jelly (trial project), food product without royal jelly (placebo)

(2) Objective: To evaluate the effects of 8 continuous weeks of consumption of a food product containing royal jelly on subjective assessment of dry eyes as well as ophthalmological examination results, and to compare these effects to those of a placebo.

(3) Subjects: Men and women aged 20 to 60 years with subjective symptoms of dry eyes

(4) Number of subjects at the start of consumption: 50 (trial product: 25 participants; placebo: 25 participants)

(5) Trial design: Randomized, double-blind, placebo-controlled, parallel-group trial

(6) Amount of functional ingredient consumed: Royal jelly 7,200 mg (raw product) per day

(7) Method of consumption: 2 capsules swallowed whole with water after meals, 3 times per day

(8) Period of consumption: 8 weeks

(9) Planned trial duration: May 2014 to March 2015

**2. Trial objective**

The objective is to evaluate the effects of 8 continuous weeks of consumption of a food product containing royal jelly on the subjective assessment of dry eyes as well as ophthalmological examination results, in men and women aged between 20 and 60 years with dry eye symptoms, and to compare these effects to those of a placebo.

**3. Trial system**

3.1. Supervising investigator

Name: Kazuo Tsubota

Institution, position: Keio University School of Medicine Department of Ophthalmology; Professor

Primary duties: Provide advice on, and check trial protocol and results.

3.2. Principal and co-principal investigators

(1) Principal investigator

Name: Sachiko Inoue

Institution: Haneginomori Eye Clinic, director

Primary duties: coordinate tasks pertaining to the trial; take management responsibility; give instructions and explanations to the subjects; obtain the subject’s consent; perform medical interviews; check for and assess adverse events; fill out case report forms; manage the examination system; provide any treatment required for adverse events.

(2) Co-principal investigator

Name: Motoko Kawashima

Institution: Keio University School of Medicine Department of Ophthalmology

Primary duties: support the principal investigator; give instructions and explanations to the subjects; perform medical interviews; check for adverse events and provide the necessary treatment.

(3) Joint researcher

Name: Shigeru Nakamura

Institution: Keio University School of Medicine Department of Ophthalmology

Primary duties: support the principal and co-principal investigators; provide advice on the trial protocol; examine and give advice on the results.

3.3. Trial institution

Name: Hanegi No Mori Eye Clinic

Address: Daita 4-26-4, Setagaya Ward, Tokyo 155-0033

Primary role: this is where the trial will be conducted; physical assessments, scientific examinations, and ophthalmological examinations will be performed; subjects will be managed; and trial system will be maintained.

3.4. Ethics committee

Name: Shirasawa Clinical Trial Center ethics committee

Chairman: Shigeru Yasuraoka

Address: Tatebayashi, Gunma Prefecture

Duties: perform screenings from epidemiological, ethical, and scientific standpoints of the trial protocol, and materials related to the trial product; provide written reports on the committee’s views.

4. Trial product

4.1. Type and name of trial product

(1) Trial product: Food product containing enzyme-treated royal jelly (RJ)

(2) Placebo: Food product with no RJ

4.2. Consumption history of trial product

This health supplement containing enzyme-treated RJ has been manufactured and sold by Yamada Bee Farm since 2008. As of 2013, more than 1 million bottles had been sold to over 100,000 customers. There have been no reports of serious adverse effects caused by consumption of this product.

4.3. Functional ingredient: RJ

4.4. Composition of trial product

| Per capsule  (approximate value) | | Trial product* | Placebo |
| --- | --- | --- | --- |
| Calories | (kcal) | 2.2 | 2.03 |
| Protein | (g) | 0.16 | 0.15x10-3 |
| Fat | (g) | 0.03 | 0.025 |
| Carbohydrates | (g) | 0.31 | 0.43 |
| Sodium | (mg) | 9.43 | 0.045 |

* Each capsule contains 1,200 mg enzyme-treated RJ (raw product)

4.5. Determination of trial product intake, method of consumption, period of consumption, functional ingredient intake.

(1) Trial product intake, method of consumption.

Two capsules are to be taken unchewed with water after meals, 3 times per day. If subjects forget to take capsules after meals, they can take 6 capsules at one time on that day. Capsules for multiple days should not be taken together. Subjects will record the times and amounts taken in daily journal entries. If they forget to take capsules, this should be recorded in that day’s entry. The subjects should not take any capsules before coming to the clinic on examination days. They can take capsules after the examinations for the fourth week and beyond.

(2) Period of consumption of the trial product.

The period will be 8 weeks (56 days).

(3) Basis for determining functional ingredient intake.

Consumption of 3 to 6 capsules of the RJ product per day was used as a benchmark, with each capsule containing 1,200 g of enzyme-treated RJ (raw product). Clear effects were observed in animal experiments at 300 mg/day, so daily intake for this trial was set at 6 capsules (equivalent to 7,200 mg). Daily intake was also determined based on amounts that were confirmed to be safe in multiple past trials involving other RJ-related products.

**5. Trial design**

Randomized, double-blind, placebo-controlled, parallel-group trial

**6. Number of subjects, allotment method**

6.1. Number of subjects

There will be 50 subjects when consumption is started—25 subjects taking the trial product and 25 subjects taking the placebo.

6.2. Allotment method

The person in charge of allotment will use random numbers to create an allocation table and assign the trial product an allocation number. The person in charge of allotment will then seal the allocation table and keep it sealed until the time comes to open it. After the analysis set and data are fixed, the person in charge of allotment will unseal the allocation table and disclose the information., In the event of a serious adverse event or emergency, the allocation table can be unsealed only to disclose the minimum amount of information required.

7. Inclusion and exclusion criteria for the subjects

People who meet the following inclusion criteria and do not fall under the exclusion criteria will be selected as the subjects.

7.1. Inclusion criteria

(1) Men and women aged between 20 and 60 years.

(2) People who are aware of having dry eyes

7.2. Exclusion criteria

(1) People whose vision when wearing corrective lenses is less than 1.0 in a vision test performed during the preliminary examination

(2) People with severe myopia (-6D or greater)

(3) People who regularly take health foods, supplements, or medical products (including eye drops) that are enriched with the effective ingredient of the trial product (RJ).

(4) People who currently have an ophthalmological disease or have a history of serious ophthalmological disease.

(5) People diagnosed with the following vision disorders: cataracts (congenital, senile, diabetic, concurrent, traumatic), glaucoma (chronic, acute, congenital, secondary).

(6) People who may experience hay fever from July to December or those diagnosed with asthma

(7) People currently undergoing pharmaceutical therapy or people with a history of a critical disease that required pharmaceutical therapy.

(8) People who underwent corrective laser eye surgery in the last 3 months.

(9) People who may have an allergy to the trial product.

(10) People who participated in a clinical trial within 1 month of agreeing to participate in this trial, or who plan to participate in another clinical trial after agreeing to participate in this trial

(11) People who plan to become pregnant or breastfeed during the trial period

(12) People who are otherwise deemed unsuitable by the principal investigator

**8. Subject consent**

8.1. Timing of obtaining consent

Consent should be obtained in writing before the first examination is performed (see examination schedule below).

8.2. Content of explanatory documents

The explanation given to the subjects should cover the following items:

・Name of trial institution, names of principal and other investigators

・Purpose, significance, methods, and duration of this trial (study)

・The voluntary nature of participation in this trial (study)

・The fact that they will not face any disadvantage for not agreeing to participate in this trial (study)

・That they can revoke their consent to participate in this trial (study) at any time

・Any potential benefits, risks, and inevitable discomforts from participating in this trial (study)

・How risks and inevitable discomforts associated with this trial (study) will be compensated for, or otherwise dealt with

・The source of funding for this trial (study), potential conflicts of interest, and relationships the investigators and others have with outside organizations

・How personal and other information will be treated

・That the results of this trial (study) may be published after ensuring the subjects cannot be identified

・Details about joint trials (studies), if any are being conducted

・Where to lodge complaints regarding the handling of personal and or other information

・Things to abide by during the trial (study)

・Delivery of new information, and changes to the trial protocol

・Costs related to the trial (study), and cooperation payments

・How materials will be stored, used, or disposed of after the trial (study) ends, (including that it may be used in other trials, and the expected content of such trials)

・About the ethics committee and publication of summaries of its meetings

8.3. Method of obtaining consent

Following the spirit of the Declaration of Helsinki, subjects will be given the latest explanatory documents and consent forms approved by the ethics committee before taking part in the trial (before the first examination). They will be given a full explanation of the trial and the investigators will strive to answer their questions satisfactorily. The subjects will be given sufficient time to ask questions and decide whether to participate in the trial. The (co-) principal investigator will obtain the consent from the subjects in writing, of their own free will and accord. The consent forms will be dated and signed (or stamped) by the subject and the (co-) principal investigator.

8.4. Delivering information that could affect the subject’s intentions, revisions to explanatory documents and consent form.

If the trial sponsor obtains new and important information that could affect a subject’s consent, it should be quickly communicated in writing to the principal investigator. If the (co-) principal investigator obtains new and important information that could affect a subject’s consent, it should be quickly communicated to the subjects to confirm they are willing to continue the trial. If the principal investigator decides the explanatory documents or consent form need to be revised based on newly obtained information, the revisions should be made quickly, then given to the participants after obtaining approval of the ethics committee to reconfirm their consent (confirm that they are willing to continue the trial).

**9. Protecting personal, and or other information**

The people involved in the trial will give full consideration to protecting a subject’s personal information and privacy when handling the consent forms and case report forms, and when publishing the data. Filling out case report forms, publishing articles, and submitting materials to regulatory authorities should be done in such a manner that a subject cannot be identified (for example assigning them identification numbers) to protect their privacy.

| Examination day | | Preliminary period |  | Consumption period (8 weeks) | | |
| --- | --- | --- | --- | --- | --- | --- |
|  | | Preliminary |  | Pre-consumption | Week 4 of consumption | Week 8 of consumption |
| Inclusion, allotment | |  | ○ |  |  |  |
| Lifestyle questionnaire | | ○ |  |  |  |  |
| Interview | | ○ |  | ○ | ○ | ○ |
| Physical measurements | Height | ○ |  |  |  |  |
|  | Weight | ○ |  | ○ | ○ | ○ |
| Scientific examinations | Systolic blood pressure | ○ |  | ○ | ○ | ○ |
|  | Diastolic blood pressure | ○ |  | ○ | ○ | ○ |
|  | Heart rate | ○ |  | ○ | ○ | ○ |
|  | Biochemical data |  |  | ○ |  | ○ |
|  | Fundoscopy | ○ |  |  |  |  |
|  | Vision test, refractometry | ○ |  |  |  |  |
|  | Functional visual acuityt |  |  | ○ | ○ | ○ |
|  | BUT | ○ |  | ○ | ○ | ○ |
|  | Keratoconjunctival disorder | ○ |  | ○ | ○ | ○ |
|  | Schirmer’s test | ○ |  | ○ | ○ | ○ |
| Subjective dry eye evaluation | DEQS | ○ |  | ○ | ○ | ○ |
| Consumption of trial product | |  |  | 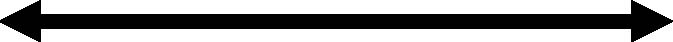 | | |
| Journal records | |  |  | 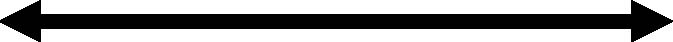 | | |

**10. Schedule and examination content**

10.1. Trial schedule

(1) Preliminary examinations will be performed on candidate subjects who give consent. These will include a lifestyle questionnaire, interview, physical measurements, scientific measurements, ophthalmological examinations ( fundoscopy, vision test, refractometry, Schirmer’s test), and subjective evaluation of dry eyes (questionnaire).

(2) Fifty people who fit the objective of the trial will be selected based on the results of the preliminary examinations.

(3) The subjects who are selected will come to the clinic for pre-consumption examinations, which will include an interview, physical measurements, scientific measurements, ophthalmological examinations (functional vision test, break-up time of tear film (BUT), keratoconjunctival disorders), and subjective evaluation of dry eyes (questionnaire).

(4) After the pre-consumption examinations, the subjects will be given the allotted trial product so that they can begin taking it the following day. They will also begin making journal entries of details such as the time they consume the trial product.

(5) The subjects will come to the clinic during the fourth and eighth weeks of the trial to undergo physical measurements, scientific measurements, and ophthalmological examinations (functional vision test, BUT, keratoconjunctival disorders, Schirmer’s test).

10.2. Examination days

The subjects will come to the clinic a total of 4 times for examinations. If a subject cannot undergo an examination on the designated day for some reason, the principal or co-principal investigator will decide on another day to perform the examination that is within 7 days before or after the designated day. If necessary, repeat examinations and follow-up examinations will be conducted.

10.3. Survey and examination content

See the schedule above.

| Item | Content | |
| --- | --- | --- |
| Lifestyle questionnaire | Medical history, medical product and health food intake, allergies, smoking status, alcohol intake, subjective dry eye symptoms (DEQS), etc. | |
| Interview | Inquire about physical condition, check for adverse events | |
| Physical measurements | Height*^1^, weight, BMI | |
| Scientific examinations | Systolic blood pressure, diastolic blood pressure, heart rate, biochemical data(total protein, aspartate aminotransferase, alanine transaminase, lactate dehydrogenase, alkaline phosphatase, gamma-glutamyl transferase, urea nitrogen, creatine, uric acid, total cholesterol, low-density lipoprotein cholesterol, high-density lipoprotein cholesterol triglyceride, glucose, haemoglobin A1c) | |
|  | Fundoscopy | Examination by physician during interview*^1^ |
|  | Vision test | Measured using a visual examination device*^1^ |
|  | Functional vision test, | Examined using a functional optometer*^2^ |
|  | Refractometry | Examined using autorefractor keratometer*1 |
|  | BUT | Fluorescein BUT*^2^ |
|  | Keratoconjunctival disorder | Fluorescein stain test*^2^ |
|  | Schirmer’s test | *^3^ |

1. ^1^: Performed at preliminary examination only
2. ^2^: Performed starting with pre-consumption examination
3. ^3^: Performed at preliminary examination and eighth-week examination

10.4. Restrictions on the subjects (precautions)

(1) Take 2 capsules of the trial product after each meal every day during the consumption period of the trial, and record this in a journal.

(2) During the trial, maintain lifestyle habits from before the trial in areas such as diet, alcohol consumption, exercise, and sleep. Avoid large deviations from the normal range for exercise and food consumption (both under and over-eating).

(3) Do not consume any new health foods or supplements during the trial.

(4) Do not drink alcohol the day before or the day of examination, until the examination is over.

(5) Do not wear contact lenses the day before or the day of examination, until the examination is over. Bring the same glasses worn for the preliminary examination to each subsequent examination.

(6) Finish designated meals 2 hours before coming to the clinic on examination days (water can be consumed).

(7) Do not smoke for at least 2 hours before coming to the clinic on examination days.

**11. Expected side effects**

At present, no side effects are expected, although general symptoms from food (abdominal pain, diarrhea, other abdominal symptoms) could occur very rarely.

**12. Adverse events**

12.1. Definition of adverse events

Adverse events are defined as unwanted medical events that appear or worsen after consuming the trial product (subjective symptoms, objective signs, abnormal changes in examination values). There need not be a causal relationship with the trial product.

12.2. Assessment of adverse events

Adverse events that occur as subjective symptoms or objective signs will be assessed by the principal investigator. Whether abnormal changes in examination values should be considered adverse events will be determined by the principal investigator, based on the reference values of the trial institution and by referencing assessment criteria from the NCI, CTC, and Japanese Society of Chemotherapy (Chemotherapy. 2010.58 (4))

12.3. Collection and recording of adverse events

When an adverse event occurs, appropriate treatment will be administered as necessary. In addition, details about the symptoms and signs (diagnosis name), severity of adverse event, whether it is critical, has a relationship to the trial product, date of appearance, date of disappearance, whether the subject can continue the trial, whether hospital treatment was given (details of treatment), outcome, and comments by the physician will be recorded on the case report form. The date of appearance is defined as the date the adverse event appeared (if unable to determine, then record the date of confirmation), and the date of disappearance is the date the adverse event disappeared (if unable to determine, date of confirmation; if it does not disappear, date the outcome was confirmed).

Adverse events will be assessed according to the following criteria.

(1) Severity

Mild: Does not disturb daily life

Moderate: Disturbs daily life

Severe: Makes daily life almost impossible

(2) Critical adverse events

If an adverse event falls under any of the following criteria, it will be considered critical.

Death

An event that could lead to death (life-threatening event)

Events that require hospitalization for treatment, or that prolong a hospitalization

Causes disability (events that cause dysfunctions that inhibit daily activities)

Events that could lead to disability

Critical events that conform to the events (disabilities) described above

Causes congenital disease or abnormalities in later generations

(3) Relationship to trial product

No: A cause that is clearly not the trial product can be identified

Probably no: No logical, chronological, or medical connection can be established with consumption of the trial product

Possibly yes: A logical, chronological, or medical connection can be established with consumption of the trial product, but other causes are also suspected

Yes: A clear logical, chronological, or medical connection can be established with consumption of the trial product, and no other cause is suspected

(4) Outcomes

Recovery, disappearance: Symptoms or findings disappear; examination values normalize or return to pre-consumption levels

Recession: Severity decreases by 1 grade or more, mild symptoms or findings nearly disappear, examination values normalize or recover to near pre-consumption levels

- - 1. No change: Nearly no change in symptoms, findings, or examination values
       1. Aggravation: Symptoms, findings, or examination values worsen

Unclear: Following up on symptoms or examination values was attempted, but was unsuccessful (includes death) (example: change of address).

(5) Continuance of trial product intake

Continue: Continuing to take the trial product (continues the trial)

Suspension: Temporarily stopping trial product intake for observation

Termination: Stopping trial product intake

12.4. Side effects

Side effects are adverse events that are determined to have or possibly have a relationship with the trial product, and for which a causal relationship with the trial product cannot be ruled out.

12.5. Follow-up examinations

When an adverse event occurs, follow up examinations will be conducted until the event disappears, a recovery trend is observed, or the principal investigator decides they are no longer necessary.

12.6. Reporting procedure when critical adverse events occur

If an adverse event is determined to be critical, the principal investigator will notify the chief of the trial institution soon after it is discovered. A report on the critical adverse event will be made and submitted to the chief of the trial institution.

**13. Termination and dropping out**

13.1 Definitions

Termination when the primary investigator decides to discontinue the trial before it is completed. Dropping out is defined as when a subject decides to discontinue the trial due to circumstances or his/her desire before the trial is completed.

13.2. Termination

If any of the following events occur, the primary investigator can discontinue a particular subject’s participation in the trial or the entire trial.

(1) Termination of a particular subject

If a subject’s safety could be at risk

If a critical clinical abnormality or accident occurs that makes continuing the trial difficult

If the subject makes serious or continuous infringements to the trial protocol

If the principal investigator decides, the trial should be terminated for any other reason

(2) Terminating the trial

If the trial sponsor needs to terminate the entire trial for some reason, the sponsor will cooperate with the contract research organizations to determine a plan for ending the trial and for what will occur afterwards. The sponsor will quickly notify in writing the primary investigator, ethics committee, and trial institution that the trial is being terminated and the reasons for this.

If the principal investigator is notified that the entire trial will be terminated, or if information is obtained that could affect a subject’s continued participation in the trial, the subjects will be provided with this information.

If the ethics committee requests anything after the trial is terminated, the trial sponsor will work with the contract research organizations and principal investigator to address this request.

13.3. Dropping out

If a subject, after agreeing to participate in the trial, decides to drop out due to circumstances or his/her desire, the principal investigator will terminate the subject’s participation in the trial.

13.4. Dealing with terminations (dropouts)

In the event of a termination (dropout), the date and reason for the termination (dropout) will be recorded in the case report form.

**14. Selecting the analysis set**

14.1. Efficacy analysis set

The analysis set will comprise subjects who complete all of the trial’s designated schedule and content, and exclude those who fall under the following criteria:

[Criteria for exclusion from the analysis set]

(1) Consuming less than 80% of the trial product

(2) Behavior that could harm the reliability of the results, such as missing journal entries

(3) Discovery that a subject falls under the exclusion criteria after he/she is included in the trial, or that a subject violated the restrictions (precautions) during the trial

(4) Other reasons that make exclusion appropriate

14.2. Safety analysis set

(1) Adverse events

Subjects who experienced an adverse event and consumed the trial product at least once

(2) Examination values

Subjects who completed all of the trial’s designated schedule and content

**15. Evaluation methods**

15.1 Evaluating efficacy

(1) Primary outcomes: subjective dry eye symptoms, BUT, keratoconjunctival disorders, Schirmer’s test

(2) Secondary outcomes: functional vision

(3) Efficacy evaluation methods

For each item, values before consumption and at each time point after consumption will be compared between the trial group and the placebo group using a 2-sample t-test. The amount of change from before consumption to each time point after consumption will be compared between the trial group and the placebo group using a 2-sample t-test. Values at each time point after consumption will be compared to values before consumption in each group using a 1-sample t-test.

For the baseline characteristics of patients with dry eye, Student’s t-test (p ≥ 0.05 by the F-test) will be used for comparison of age, and Pearson chi-square test will be used for categorical data of gender and dry eye diagnosis. Repeated measures analysis of variance followed by Tukey's honest significant difference (HSD) post hoc test will be used for analysis of dry eye symptoms. For the biochemical analysis, paired t-tests will be used for within-group comparisons and the F-test, followed by Student’s t-test (p≥0.05 by the F-test) will be used for between-group comparisons (placebo vs. RJ). A p-value of <0.05 was considered statistically significant. 16.2. Evaluating safety

(1) Outcomes

These are adverse events confirmed from interviews and journal entries (subjective symptoms), and abnormal variations in body weight and scientific examination values. For examination values, changes in items will be checked: body weight, systolic blood pressure, diastolic blood pressure, heart rate, and biochemical data.

(2) Evaluation methods

The number of adverse events and abnormal variations will be totaled to create a table. If the same adverse event occurs in the same subject multiple times, it will be treated as one event. To evaluate changes in examination values, measurements before consumption and at each time point after consumption will be compared using a 1-sample t-test. Figures will be displayed as mean ± standard deviation (or standard error). The significance level will be 2-sided 5%.

16.3. Compliance with, changes to, deviations from, or revisions to the trail protocol

The primary investigator will comply with the trial protocol and not deviate from, or change it without first obtaining the written consent of the trial sponsor. Moreover, if the trial protocol is changed after being approved by the ethics committee, the changes will need to be reapproved by the ethics committee.

However, this does not apply to a situations involving urgent danger to a subject, scenario’s that are medically unavoidable, or circumstances related to clerical matters (example: changing a phone number).

**17. Filling out case report forms**

The primary investigator will fill out a case report form on each subject’s results. These will be signed or stamped, and then submitted to the trial sponsor. Data on the case report form should not contradict the original materials. In addition, personal information should not be included without the subject’s consent.

**18. Ethics**

18.1. Subject selection

The following areas will be considered when selecting the subjects: human rights protections; the trial protocol’s inclusion and exclusion criteria; the subject’s health status, age, sex, ability to give consent, dependence on the principal investigator, and participation in other trials; and whether it is appropriate to ask the subject to participate in this trial.

People who lack the ability to provide consent will not be selected as subjects. When selecting people who could experience some kind of unfair disadvantage from not participating in the trial, full consideration will be given to ensure their consent is being given voluntarily.

18.2. Declaration of Helsinki and ethical guidelines for epidemiological research

This trial will be conducted by following the spirit of the Declaration of Helsinki and according to ethical guidelines for epidemiological research.

18.3. Dealing with subjects whose health is harmed

If a subject’s health is harmed by participating in this trial, the principal investigator will provide whatever therapies or treatments are necessary. However, this does not apply to damage caused by intentionally by a subject or one that is secondary to their mistake. The trial sponsor will obtain insurance and other necessary measures so that they can fulfill their responsibilities.

18.4. Explanation of results

After the trial ends, the subjects may obtain an explanation of the examination and other results from the principal or co-principal investigator.

**19. Viewing original materials**

If a person in charge of the matter from the trial sponsor wishes to check the surveys or other records, the principal investigator and trial institution will allow the surveys and original materials (original copies of consent forms, journals and other records submitted by the subjects, data recorded on case report forms) to be viewed. However, privacy protection will be given full consideration when viewing original materials that contain personal information.

**20. Payments**

21.1. Costs of the trial

This trial will be paid for by a research grant (Yamada Bee Farm research support fund).

21.2. Cooperation payments

The subjects will be given cooperation payments calculated based on the rules of the trial institution.

**21. Storage of records**

21.1. Chief of the trial institution

The chief of the trial institution will store all documents related to the trial for 5 years after the trial is terminated or ends. These documents include original materials (original copies of consent forms, journals and other records submitted by subjects, data recorded on case report forms), contracts, documents related to ethics committee screenings, and other clerical documents related to the trial, such as records on managing the trial product. However, if the trial sponsor requires a longer storage period, the storage duration and methods will be discussed. Moreover, if the trial institution has rules concerning the storage of records, these rules will be followed.

21.2. Principal investigator

The principal investigator will store all records related to the trial according to instructions given by the chief of the trial institution.

**22. Disposal of records**

Once the storage period has passed, consent forms and other materials signed by the subjects will be disposed of properly (incineration, etc.). Anonymized records can be disposed of as is (incineration, etc.).

**23. Planned trial duration**

May 2014 to March 2015

End
